# Role of microRNAs in glioblastoma

**DOI:** 10.18632/oncotarget.28039

**Published:** 2021-08-17

**Authors:** Ming Chen, Zdravka Medarova, Anna Moore

**Affiliations:** ^1^Precision Health Program, Michigan State University, East Lansing, MI 48824, USA; ^2^Department of Radiology, College of Human Medicine, Michigan State University, East Lansing, MI 48824, USA; ^3^Athinoula A. Martinos Center for Biomedical Imaging, Department of Radiology, Massachusetts General Hospital and Harvard Medical School, Charlestown, MA 02129, USA

**Keywords:** glioblastoma, microRNA, miRNA-based therapies, miRNA delivery

## Abstract

Glioblastoma is the most common and aggressive primary human brain cancer. MicroRNAs (miRNAs) are a set of small endogenous non-coding RNA molecules which play critical roles in different biological processes including cancer. The realization of miRNA regulatory functions in GBM has demonstrated that these molecules play a critical role in its initiation, progression and response to therapy. In this review we discuss the studies related to miRNA discovery and function in glioblastoma. We first summarize the typical miRNAs and their roles in GBM. Then we debate the potential for miRNA-based therapy for glioblastoma, including various delivery strategies. We surmise that future directions identified by these studies will point towards the necessity for therapeutic development and optimization to improve the outcomes for patients with glioblastoma.

## INTRODUCTION

Glioblastoma (also known as glioblastoma multiforme, GBM) is the most aggressive primary brain cancer in humans [1] with a median survival time of 15 months and a 5-year survival rate of only 10% [2–4]. Traditional treatments such as surgery, chemotherapy, and radiation therapy do not work well for GBM. Surgery cannot completely remove GBM tumors because of the complex structure of the brain and infiltration of tumor cells into brain tissues. Furthermore, radiation therapy and chemotherapy are not very effective over the long term due to radio- and chemoresistance. GBM may initially respond to radiotherapy and chemotherapy, however, subsequent local recurrence is very common. Therefore, GBM is considered incurable and traditional treatments can only extend survival time by months to years depending on age, performance status, extent of resection and molecular features [5].

In the last several decades, scientists have had a great interest in improving treatment outcomes and, as a result, extensive progress has been made. However, there are still some significant biological issues that need to be overcome in order to achieve tangible clinical success. These basic biologic difficulties include GBM tumor location, tumor heterogeneity, and the blood-brain barrier (BBB). Tumor location is critical when considering surgical resection. Due to the brain stem, eloquent cortex, and the infiltration of the tumor into surrounding tissue, total surgical removal of a tumor mass represents a significant risk. Tumor heterogeneity is also a characteristic feature of glioblastoma, which has been thoroughly investigated. Extensive cellular and genetic heterogeneity in GBM has been found not only between patients (inter-tumoral), but also at an intra-tumoral level [6–8]. Recent studies demonstrated that multiple factors contribute to this heterogeneity including multiple subtypes of glioblastoma stem cells (GSC) some of which are highly invasive and could contribute to disease’s rapid progression [9]. A recent study published in Cell [10] applied a single-cell transcriptome analysis of patient samples and revealed significant cell-type heterogeneity in their molecular signatures. Glioblastoma 3D organoids generated in this study can recapitulate inter- and intra-tumoral heterogeneity and retain many key features of their corresponding parental tumors, which will help in devising new therapies on a clinically relevant timescale [10].

All of these features are critical determinants of chemotherapeutic efficacy. For example, temozolomide (TMZ), an FDA approved chemotherapeutic for GBM, can generate drug-resistant clones under long-term exposure due to the cellular heterogeneity of the tumor.

The BBB is also a major feature of brain cancer. It is formed by brain microvascular endothelial cells that are sealed by tight junctions. Most chemotherapeutics are not able to cross the blood brain barrier and reach the tumor site [11]. There are multiple reasons contributing to inability of chemotherapeutics to reach the tumor. The BBB has a number of highly selective mechanisms for transporting substances into the brain [12–18]. Brain endothelial cells are closely connected to form tight junctions. Only some very small lipophilic molecules or small gas molecules (such as CO_2_, or O_2_) can freely and passively diffuse through the BBB via paracellular (between cells) trafficking in between the endothelial cells [19]. Active transport happens through protein carriers (transporters) with specific binding sites that undergo a change in affinity. Active transport requires ATP hydrolysis which generates energy and conducts movement against the concentration gradient. For example, GLUT-1, large neutral amino acid transporters (LAT), nucleoside transporters and also organic cation and anion transporters have been shown to play a key role for maintaining the high metabolic needs of the brain [20–23]. Another significant transport mechanism at the BBB is carrier-mediated efflux. ATP-binding cassette (ABC) transporters are ATP-driven drug efflux pumps which excrete neurotoxic substances such as P-glycoprotein, breast cancer resistance proteins (BCRP/ABCG2) and members of the multidrug resistance related proteins (MRP1, 2, 4 and 5, ABCC) in BBB [20, 24–27]. Because of their ability to transport a broad range of compounds, these efflux proteins cause a major obstacle for therapeutic delivery to brain tumors [28].

Recent studies have also shown that aberrant gene expression in glioblastoma is associated with tumorigenesis and progression not only in tumor cells but also in endothelial cells. Hupe et al. used translational profiling to identify factors that are involved in BBB development and found that expression of *Foxf2* and *Zic3* gene in human umbilical vein endothelial cells induced the production of BBB differentiation markers and affected the maturation of the BBB [29]. Urich et al. showed that the expression of claudin-5, occludin and JAM2 genes in human brain endothelial cell lines relate to their low transcellular electric resistance and paracellular leakiness, low levels of unique brain endothelial transporters such as Glut1 and Pgp, and cell surface receptors such as LRP1, RAGE and the insulin receptor [30].

Due to these limitations and BBB complex physiology, the prognosis of GBM patients is poor representing an unmet clinical need in novel therapeutic approaches, such as stem cell therapy, gene therapy, immunotherapy, and miRNA therapy, which may have the potential to overcome these issues and lead to better treatment.

**Figure 1:**
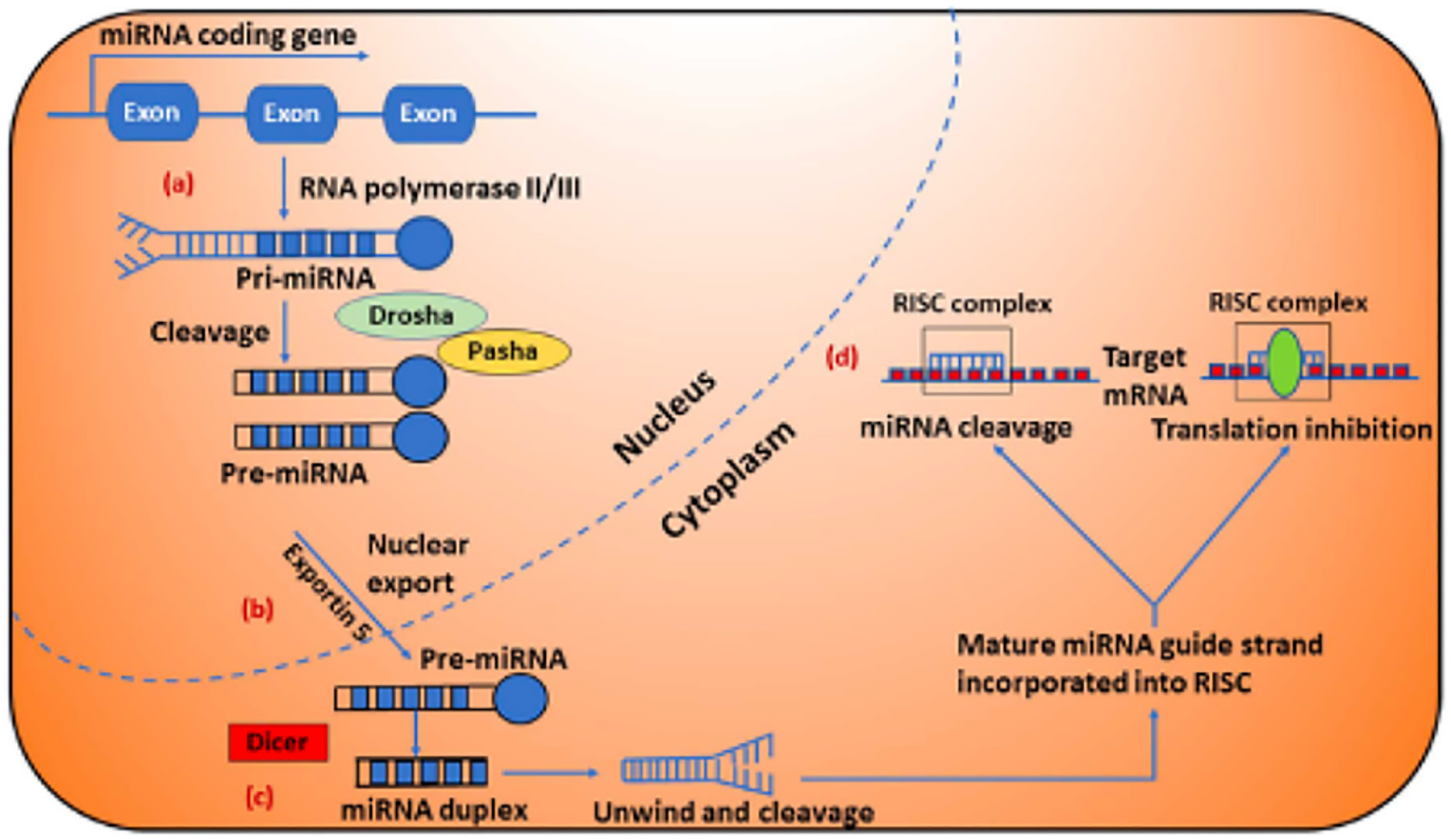
Biogenesis and clinical implications of microRNAs (miRNAs). (**A**) miRNA genes are typically transcribed by RNA polymerase-II and produce long primary miRNA (pri-miRNA), which are recognized and cleaved in the nucleus by the RNA polymerase III enzyme Drosha. (**B**) Next, pri-miRNA is processed to precursor miRNA (pre-miRNA) hairpin like structure in the nucleus by the Drosha/Pasha complex, are then transported into the cytoplasm by Exportin 5 and further is processed by another RNase enzyme called Dicer, produce miRNA duplexes. (**C**) The miRNA duplexes (miRNA:miRNA^*^ duplexes shown in blue color) are then unwound and the guide strands are selected by Argonaute for integration into the RNA induced silencing complex (RISC). (**D**) The mature miRNA leads RISC to cleave the mRNA or induce translational repression depending on the degree of the miRNA and its target genes. Figure was reproduced with permission of Dr. Ahir [31].

MicroRNAs (miRNAs) are a set of small (19–22 nucleotide long), endogenous, non-coding RNA molecules. While miRNAs cannot be translated into protein, they bind to the 3’ untranslated region (UTR) of target mRNAs and inhibit mRNA stability or translation. Biogenesis and clinical implications of microRNAs are shown in Figure 1 [31]. miRNAs regulate cell functions by either repressing transcription or inducing mRNA degradation. miRNAs have proven involvement in different cellular functions, such as proliferation, migration, differentiation, and apoptosis [32]. miRNAs have also been shown to relate to tumorigenesis, including invasiveness, DNA repair, and acquired resistance among others. These functions of miRNAs have a potential to advance this class of molecules as not only biomarkers but also therapeutic targets in GBM [33]. Depending on miRNA expression level, there is a need to either downregulate them by delivering RNA inhibitors or upregulate them by delivering miRNA mimics. Either way, an RNA molecule needs to be delivered to the cell of interest. Challenges that surround this delivery include but not limited to degradation by nucleases in biological environment [34, 35], poor ability to penetrate cell membrane because of the negative charge [36], entrapment in the endosome compartment [37], poor binding affinity for complementary sequences [38], poor delivery efficacy to target tissues [37], off-target and toxic effects and activation of immune responses [39].

In this review, we summarize general aspects of miRNAs in GBM focusing on their role in carcinogenesis and the potential for miRNA-based therapy including delivery strategies.

### GBM-related microRNAs

In the past two decades, scientists have shown that miRNAs play critical roles in human cancer. The realization of miRNA’s regulatory functions in GBM has stimulated significant body of research. In 2005, Ciafrè et al. used the microarray technique to screen the expression levels of 245 miRNAs in GBM [40]. Later, after investigating 256 miRNAs in GBM Møller et al. found that miRNAs could be either overexpressed or underexpressed [41]. Downregulation of miRNA expression was also observed in GBM tissues and cell lines in another study [31]. Concurrently, miRNAs were proven to be important regulators of gene expression and actively involved in modulating many cellular processes including apoptosis, proliferation, invasion, angiogenesis, and chemoresistance [2]. According to the roles that miRNAs play in tumorigenesis, they are classified into either tumor suppressor or oncogenic miRNAs. However, there are some miRNAs which can act both as tumor suppressors and as oncogenes depending on the tissue and tumor types [42]. The typical miRNAs found in GBM are listed in Table 1.

**Table 1:** Abundant miRNAs and their functions in GBM

miRNA	Role in GBM	Functions	Targets	Reference
miR-7	Tumor suppressor	Survival, proliferation, apoptosis, invasion, angiogenesis	FAK, EGFR, Akt, c-KIT, TGFβ2, CDK6, AKT2, LRRC4, YBX1, CD24, and MTDH	[43–48, 50, 173]
miR-34a	Tumor suppressor	Survival, proliferation, apoptosis, migration, invasion, stemness	SIRT1, c-Met, Notch1/2, PDGFRA, Msi1, Akt and Wnt	[51–60]
miR-128	Tumor suppressor	Proliferation, apoptosis, angiogenesis, stemness, radioresistance	P70S6K1, SUZ12, BMI1, PDGFRα, EGFR, E2F3a, WEE1 and Msi1	[43, 61–68]
miR-10b	oncomiR	Proliferation, apoptosis, migration, invasion, stemness	HOXD10, uPAR, RhoC, PTEN, BCL2L11, TFAP2C, CDKN1A and CDKN2A	[40, 43, 69–74]
miR-21	oncomiR	Survival, proliferation, apoptosis, migration, invasion, chemoresistance	HNRPK, TAp63, PDCD4, P53, TGF-β, MMPs, Ras/Raf, ERK, ANP32A, SMARCA4, PTEN, SPRY2, and LRRFIP1	[40, 44, 75–91, 93–95]
miR-93	oncomiR	Survival, proliferation, angiogenesis, stemness	Integrin b8	[69, 96–102]

### Tumor suppressor microRNAs

There are several miRNAs that target oncogenes and play tumor suppressive roles in GBM. When these tumor suppressor miRNAs are overexpressed, they inhibit tumorigenesis and tumor progression. The well-studied tumor suppressor microRNAs miR-7, miR-34a and miR-128 are discussed below.

### miR-7

miR-7 is downregulated in GBM [43–48]. It is one of the most potent tumor suppressors in glioblastoma and has been shown to regulate proliferation, migration/invasion, and metastasis. Liu et al. showed that miR-7 can target multiple oncogenes (such as PI3K and Raf-1) via the EGFR pathway, providing mechanistic insight into the role of this miRNA in tumor cell proliferation and viability [49]. These effects translated into a significant inhibition of glioblastoma xenograft growth *in vivo* [49]. In another study, Kefas et al. found that miR-7 inhibited the EGFR and Akt pathways in GBM, and transfection with miR-7 decreased GBM cell viability and invasiveness [48]. Recently, Wu et al. found a relationship between miR-7 and targeting focal adhesion kinase (FAK). They showed that by targeting FAK, miR-7 reduced the expression of MMP2 and MMP9 and inhibited the ability of GBM cells to migrate through the extracellular matrix (ECM) [46]. In addition, miR-7 targets c-KIT, TGFβ2, CDK6, AKT2, LRRC4, YBX1, CD24, and MTDH, and may also regulate neuronal differentiation and brain tumorigenesis [50]. All of this evidence implies that miR-7 may be a key factor and a potential therapeutic target in GBM.

### miR-34a

miR-34a is another tumor suppressor miRNA which is downregulated in GBM [51, 52]. miR-34a is one of the p53 target genes and forms a positive feedback loop with p53. Luan et al. found a connection between the level of miR-34a and the status of p53, and that miR-34a regulated p53 expression by targeting SIRT1 [53]. Li et al. showed that miR-34a inhibited brain tumor growth by downregulating c-Met and Notch [51]. In addition, Guessous et al. demonstrated that overexpression of miR-34a promoted glioma stem cell differentiation and apoptosis *in vivo* [54]. Overexpression of miR-34a also induced apoptosis in other GBM cell lines [55, 56]. Yin et al. showed that increasing the level of miR-34a in GBM cells inhibited their migration, proliferation, and angiogenesis. These researchers also found that miR-34a decreased the expression of EGFR and lowered the levels of proteins related to cell proliferation [52]. Rathod et al. demonstrated that miR-34a regulated glioma stem cell proliferation, invasion, apoptosis, and cell cycle arrest through the Akt and Wnt pathways [57]. Fan et al. showed that miR-34a mimics could trigger cell death in p53 mutant and chemoresistant GBM cell lines [58]. Recently, additional new miR-34a targets (such as Musashi1 and platelet-derived growth factor receptor-α) have been identified in GBM [59, 60]. All of these findings indicate that miR-34a could be a potential therapeutic agent for GBM.

### miR-128

Like other tumor suppressor microRNAs, miR-128 expression is also downregulated in GBM [61]. It has been reported that miR-128 inhibits tumor growth through multiple targets in many GBM cell lines. Godlewski et al. found that high levels of miR-128 inhibited glioma cell proliferation *in vitro* and xenograft tumor growth *in vivo* via direct regulation of the Bmi-1 gene [62]. Mechanistically, this effect of miR-128 in GBM was linked to inhibition of self-renewal of glioma stem cells (GSCs) via the Bmi-1 pathway. This was the first demonstration of a strong connection between miRNA and stem cell properties in GBM. Furthermore, miR-128 was found to reduce glioma cell proliferation by targeting E2F3a [63, 64]. Shan et al. also proved that miR-128 inhibited GBM and glioma stem-like cell proliferation, invasion, and self-renewal via the BMI1 and E2F3 pathways [65]. Papagiannakopoulos et al. found that miR-128 decreased gliomagenesis by downregulating growth factor receptors EGFR and PDGFRA [66]. Besides EGFR and PDGFRA [66] miR-128 was found to inhibit GBM cell proliferation via targets such as WEE1 [43], MSI1, and E2F3A [63]. Bhaskaran et al. further demonstrated that survival was significantly increased in intracranial GBM murine models by co-administration of miR-128 and the other miRNAs [67]. miR-128 was also found to regulate angiogenesis by inhibiting P70S6K1 kinase [68]. Shi et al. found that upregulation of miR-128 attenuated the effects of cell proliferation, tumor growth and angiogenesis [68]. These data support the notion that miR-128 plays a critical role in repressing GBM growth and invasion.

### OncomiRs

Although most miRNAs have tumor suppressive roles in GBM, there are some miRNAs (oncomiRs) which are upregulated in GBM and target the expression of tumor suppressor genes to promote oncogenesis. Here, the most important oncomiRs in GBM including miR-10b, miR-21 and miR-93 are discussed.

### miR-10b

miR-10b has been extensively studied in GBM [40, 43, 69–72]. Overexpression of miR-10b has been observed in higher grade gliomas, providing evidence of its relevance to clinical GBM [70, 71]. miR-10b has multiple targets. It was found that RhoC and uPAR were directly proportional to the level of miR-10b, thereby enhancing the invasive capabilities of high-grade glioma [70]. In addition to uPAR and RhoC, HOXD10 has also been identified as a direct miR-10b target [71]. Sun et al. showed that by its influence on these targets, inhibition of miR-10b resulted in reducing cell growth, invasion, and angiogenesis, as well as increasing apoptosis in GBM [71]. It was found that the direct targets of miR-10b related to cell growth were BCL2L11, TFAP2C, CDKN1A, CDKN2A, etc. [72]. Inhibition of miR-10b restored the expression of these gene targets and decreased the growth of glioma cells through apoptosis and/or cell cycle arrest. Teplyuk et al. found that the effects of miR-10b were cell line dependent. miR-10b repressed E2F1 and caused cell cycle arrest in p21-high cell lines, but not in p21-low cell lines [73]. Guessous et al. showed that miR-10b was upregulated in both human GBM and GMB stem cell lines and inhibition of miR-10b by using the antisense approach significantly reduced the proliferation and decreased their invasion and migration. Moreover, *in vivo* studies also confirmed the inhibitory effect on the growth of stem cell-derived orthotopic GBM xenografts [74]. Overall, miR-10b is highly oncogenic in GBM suggesting that it may regulate tumorigenesis and serve as a useful target in GBM therapy.

### miR-21

The function of miR-21 in GBM has been widely investigated. miR-21 has been shown to influence cell invasion, metastasis, and resistance to chemotherapeutics [40, 44, 75–87]. Numerous studies have identified miR-21 as an apoptotic regulator. Chan et al. found that miR-21 is highly expressed in GBM cells. Knockdown of miR-21 promoted cell apoptosis via caspase activation [75]. Since then, many researchers found that the apoptosis caused by miR-21 inhibition was regulated by targeting HNRPK, TAp63, and PDCD4 [82, 84, 88]. Additional targets include P53, TGF-β, and the mitochondrial apoptotic pathways [78, 89, 90]. miR-21 has also been shown to regulate apoptosis via FASL [91]. Other oncogenic effects of miR-21, such as proliferation, invasiveness, and chemoresistance in a variety of GBM cell lines have also been reported [79, 81, 84]. ANP32A, SMARCA4, PTEN, SPRY2, and LRRFIP1 have been identified as direct target genes of miR-21 related to cell proliferation [79, 83, 85, 87]. Zhou et al. showed that inhibition of miR-21 reduced EGFR and Akt activities in GBM [83]. GBM cell growth inhibition was partially dependent on the PTEN status. By targeting MMPs, Ras/Raf, and ERK, miR-21 increases the invasive potential of GBM cells [77, 85, 87]. Treatment with antisense miR-21 oligonucleotides decreased the expression of RECK and TIMP3, thereby inhibiting the migration and invasion of GBM cells [77]. miR-21 also acts as a critical agent in drug resistance. Shi et al. showed that high expression of miR-21 reduced the effects of TMZ in U87MG cells by inhibiting the expression of Bax/Bcl-2 and caspase-3 [81]. In other studies, researchers found that inhibition of miR-21 sensitized human GBM cells to chemotherapy drugs including paclitaxel, doxorubicin, sunitinib, VM-26, and TMZ [79, 80, 92–95]. These broad effects of miR-21 on cell proliferation, apoptosis, and invasion suggest a rationale for targeting this miRNA in the treatment of GBM.

### miR-93

There is evidence that miR-93 is also a critical target in GBM. miR-93 was found to be upregulated in GBM by many research groups [69, 96–100]. miR-93 regulates different glioma cell functions such as proliferation, migration, invasion, cell cycle arrest, and chemoresistance by targeting P21 [101]. Studies by Huang et al. demonstrated that miR-93 controlled autophagic activities in GSCs by inhibiting BECN1/Beclin 1, ATG5, ATG4B, and SQSTM1/p62 [97]. Fang et al. showed that miR-93 regulates GBM cell viability, tumor growth, and vasculogenesis [102]. In particular, miR-93 enhanced blood vessel formation by targeting integrin-β8 [102]. Overexpression of miR-93 enhanced vasculogenesis in a coculture of human glioblastoma U87 cells and endothelial cells. The coculture promoted endothelial cell proliferation and blood tube formation *in vitro* and significantly induced blood vessel formation *in vivo* [102]. Integrin-β8 was also identified as one of the miR-93 direct targets. The same group also showed that miR-93 promoted blood vessel formation in GBM xenograft tumors [102]. These aspects of miR-93 function make this miRNA of particular interest for the treatment of neoangiogenesis in GBM.

### Other miRNA targets in GBM

In addition to the miRNAs discussed above there are additional targets with roles in apoptosis, angiogenesis, drug resistance, and stemness.

### Apoptosis

miR-221/222 are targets overexpressed in GBM, which regulate apoptosis by targeting PUMA. PUMA binds to Bcl-2 and Bcl-x and causes cell death. Consistent with this mechanism, miR-221/222 inhibition has been found to promote apoptosis and inhibit tumor growth [103, 104]. miR-221/222 also directly targets P27 and P57 [105]. When treated with antagomirs against miR-221/222, U251 GBM cells underwent G1/S cell cycle arrest [106]. Treatment with antagomirs against miR-221/222 has been found to induce apoptosis and sensitize human glioblastoma cells to TMZ and to radiotherapy [104, 107]. Another microRNA with an oncogenic role in GBM is miR-335. Inhibition of miR-335 significantly promoted astrocytoma apoptosis both *in vitro* and *in vivo*. Shu et al. demonstrated that when C6 cells were treated with a miR-335 antagonist, the cells underwent growth arrest, apoptosis, and invasion both *in vitro* and *in vivo* [108]. These researchers further showed that miR-335 could directly target Daam1 [108]. miRNAs with tumor-suppressive functions include miR-218 and miR-451. miR-218 was reported to reduce the expression of CDK6, therefore decreasing glioma cell proliferation and promoting apoptosis [109]. It was also found that miR-218 stimulates apoptosis via regulating EGFR and ECOP, which inhibits NFκB, thus inducing cell death [110]. With regard to miR-451, exogenous administration of miR-451 reduced cell proliferation, decreased invasion, induced cell cycle arrest, and promoted apoptosis of GBM cells [111].

### Angiogenesis

There is a class of miRNAs defined as angiomiRs. AngiomiRs have important functions in GBM neovascularization [112]. miR-125b is a key angiomiR downregulated in GBM-related endothelial cells. Smits et al. demonstrated that downregulation of miR-125b in GBM cells induced tumor vascularization [113]. miR-296 is another angiomiR which is highly expression in endothelial cells [114]. Würdinger et al. showed that high expression of miR-296 promoted endothelial cell tube formation and induced vascularization of tumors, while low expression of miR-296 decreased angiogenesis [114]. Within GBM tumors, hypoxia can also induce angiogenesis by modulating miRNA expression. For example, hypoxia regulates the level of miR-210-3p. By targeting HIF3A, overexpression of miR-210-3p stimulates HIF, VEGF, and CA9 activity, inducing vasculogenesis [115].

### Drug resistance

By using miRNA microarrays, Ujifuku et al. screened the expression of miRNAs in GBM cell lines and reported that miR-195, miR-455-3p, and miR-10a∗ were the three highly expressed miRNAs in temozolomide-resistant cells [116]. Additionally, they showed that downregulation of miR-195 can significantly increase the sensitivity to TMZ [116]. Slaby et al. also demonstrated that miR-181b and miR-181c sensitized glioblastoma cells to a radio/chemotherapy by regulating MGMT levels [117]. ABCG2 is a target gene for miRNA-328 in GBM. miR-328 inhibits the expression of ABCG2 and sensitizes glioblastoma cells to the anticancer drugs (including mitoxantrone and doxorubicin) [118]. Thus, combination of miR-328 therapy with radiation or chemotherapy may be an effective strategy for GBM treatment [119].

### Stemness

Several miRNAs including miR-128, miR-124, miR-137 [120], miR-34a [51, 54], and miR-326 [121] play important roles in glioblastoma stem cells. By analyzing differential miRNA expression in GBM stem (CD133+) and non-stem (CD133−) cells, Gal et al. found that co-administration of miR-451 and imatinib mesylate inhibited tumor development of GSCs by decreasing Myc expression only in CD133+ cells [122]. Furthermore, Yang et al. showed that miR-29a inhibits GSCs cell proliferation and tumor growth via the PDGF pathway [123]. Bier et al. identified RTVP-1 as a direct target of miR-137 and demonstrated that miR-137 inhibited the self-renewal of GSCs [124]. Yang et al. also showed that PU-PEI-mediated miR145 delivery to GBM CD133(+) cells repressed their tumorigenic and GSC-like abilities and promoted their differentiation into CD133(−)-non-GSCs [125]. These studies support the idea that miRNAs may distinctively and concertedly act together to modulate key GSC properties.

### MicroRNA-based therapies for GBM

**Figure 2:**
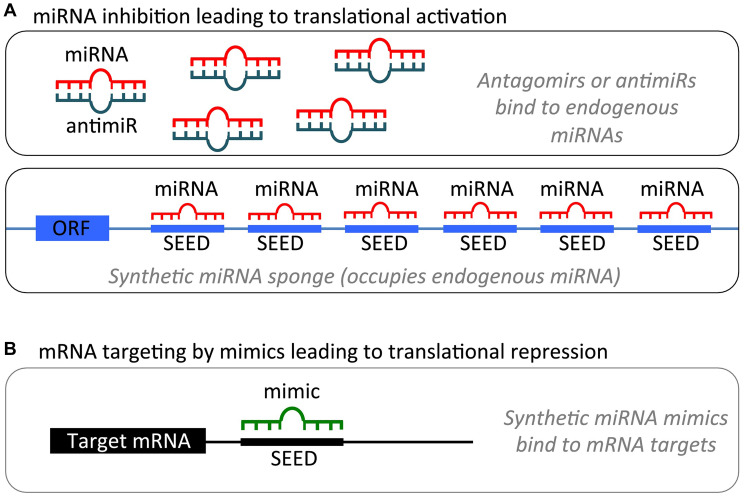
miRNA modulation strategies for therapeutic intervention. (**A**). miRNA inhibition. (1) Antagomirs are synthetic, single-stranded RNA-based oligonucleotides that are complementary to mature endogenous miRNAs, allowing for binding and silencing of their targets. (2) miRNA sponges contain multiple binding sites to an miRNA of interest, competitively inhibiting it from binding to its target mRNA. As the binding sites are specific to an miRNA’s seed region, sponges can inhibit an entire family of related miRNAs. (**B**) miRNA mimics are synthetic, double-stranded RNA molecules that have identical sequences to their naturally occurring equivalents, allowing for restoration or amplification of the activity of a target miRNA. Figure was reproduced with permission of Dr. Kumar [2].

Due to their ability to target multiple genes, miRNAs have evolved as promising therapeutic targets. As detailed above, miRNAs work as either tumor suppressors or oncomiRs and play critical roles in cell differentiation, proliferation, and apoptosis. According to their cellular functions, two fundamental strategies for miRNA-based therapy have been proposed. We can either restore the downregulated tumor suppressor miRNAs by using microRNA mimics or inhibit the overexpressed oncomiRs by using microRNA inhibitors. miRNA modulation strategies for therapeutic intervention are illustrated in Figure 2 [2].

### microRNA mimic therapy

Tumor suppressor miRNAs are always downregulated in GBM. To normalize their expression profile, the miRNA-based replacement therapy can be used to increase the expression of a given tumor suppressor molecule. The exogenous oligonucleotides (also known as miRNA mimics) which have the same sequence as the corresponding endogenous miRNAs are synthesized and delivered to GBM cells. The restoration of the tumor suppressive actions of these miRNAs inhibits cancer progression. Several preclinical trials have shown that miRNA mimics strongly inhibit GBM growth. For example, miR-34a is a well-defined tumor suppressor miRNA and is downregulated in GBM [58]. Cell death was induced by using miR-34a mimics in p53-mutant, chemoresistant GBM cells. This suggested that miR-34a mimics can be used as a novel therapeutic agent [58]. Chen et al. showed that miR-203 mimics, when transfected into U251 cells, significantly decreased the level of phospholipase D2, which is a target of miR-203. This led to the inhibition of the proliferation and invasion of U251 cells, underscoring the benefits of these miRNA mimics as therapeutics [126]. By using a polyethylenimine (PEI)-mediated delivery method, Ibrahim et al. successfully delivered miR-145 and miR-33a mimics into mouse xenograft tumors and demonstrated their antitumor effects [127]. Another delivery method known as liposome-mediated delivery of miR-34a mimic (MRX34) has been used to treat liver cancer and began phase I clinical trials in 2013 [128, 129]. However, this clinical trial was terminated in 2016 due to serious immune responses [130]. It has not been used for treatment of GBM.

### microRNA inhibitor therapy

miRNA inhibition therapy is used to inhibit tumor-promoting oncomiRs in GBM. Multiple mechanisms have been investigated recently. All have the potential to be translated into clinical practice. Each has unique pros and cons discussed below.

### Antisense oligonucleotides

Antisense oligonucleotides (termed antagomiRs or antimiRs) are synthetically produced oligonucleotides that inhibit the level of upregulated miRNAs by blocking the interaction between miRNA and its target mRNAs. Song et al. used R3V6 peptide-coupled antagomirs to inhibit miR-21. The peptide protected the oligonucleotides from cleavage by nucleases and also enhanced delivery. The conjugate was found to reduce the expression of miR-21 and promote apoptosis in GBM cells. This implies that the R3V6 peptide may serve as a powerful tool for delivery of antisense oligonucleotides [131]. Oh et al. showed that anti-miR-21 antisense oligodeoxynucleotides were delivered by R3V6 peptide *in vivo*. Apoptosis of tumor cells was strongly promoted, resulting in the effective suppression of tumor growth [132]. By using 2′-O-methyl (OMe) antisense oligonucleotide, Zhou et al. successfully induced apoptosis in GBM by inhibiting the expression level of miR-21 [82]. The LNA against miR-122 (Miraversen), a locked nucleic acid-modified DNA phosphorothioate antisense oligonucleotide, has started phase II clinical trials displaying encouraging results in patients with hepatitis C [133], however, studies in GBM have not been conducted yet.

### miRNA sponges

Similar to miRNA inhibitors, miRNA sponges are longer nucleic acids, such as DNA plasmids or transcribed RNA. miRNA sponges inhibit miRNA function by blocking a whole family of related miRNAs [134]. The miRNA sponges are transcribed from expression vectors delivered into tumor cells [134]. Chen et al. showed that a miR-23b sponge in GBM inhibited migration, invasion, and tumor progression *in vivo* [135]. Circular RNAs (circRNA) are natural miRNA sponges. There are large quantities of these RNAs in mammals. Hansen et al. showed that ciRS-7 and miR-7 were both overexpressed in the brain. The ciRS-7 sponge strongly inhibited the expression of miR-7, which led to enhanced expression of miR-7 targeted transcripts. The same researchers showed that circRNA acting as a miRNA sponge was also a very common phenomenon [136]. miRNA sponges reduce the downstream effects of its targeted miRNA. This property makes them a powerful tool for researching miRNA function *in vitro*. However, highly abundant exogenous nucleic acids may cause toxicity and off-target effects. These safety concerns make it less likely for miRNA sponges to succeed as therapeutic agents [129].

### microRNA-based drug delivery to GBM

There are several challenges to deliver active miRNA-based therapeutic agents to GBM. In order to get efficient delivery, new strategies have been developed. These include viral and non-viral delivery systems.

### Viral delivery

Viruses are widely used to effectively deliver miRNAs into tumor cells [137]. For example, Dong et al. using lentiviral vectors to deliver miR-7-3 to U251 cells, demonstrated significant inhibition of proliferation and cell cycle arrest [138]. In order to downregulate the expression of miR-10, Fatimy et al. successfully delivered CRISPR/cas9 construct *in vitro* and *in vivo* using a lentiviral vector [139]. Due to its safe and high transduction efficiency, adeno-associated viruses (AAVs) have become an attractive candidate for miRNA delivery. In a pioneering study by Kota et al., miR-26a carried by AAVs was systemically delivered to hepatocellular carcinoma (HCC) cells, resulting in cell cycle arrest, increased apoptosis and reduced tumor growth [140] representing a potential for other cancers such as GBM. Recombinant AAVs (rAAVs) are another class of promising viral vectors for delivery of therapeutic miRNAs in GBM because of low risk of triggering the host immune response [141]. However, there are still many drawbacks limiting the clinical application of viral miRNA delivery. Unfavorable side effects and challenging scale-up processes are the two critical concerns. The side effects include immunotoxicity, inflammatory response and tissue degeneration induced by immunogenicity, and mutations due to the inserted sequence [142]. Compared with non-viral delivery systems, viral miRNA delivery systems are more difficult to scale up hindering manufacturing and quality control. Therefore, non-viral-based systems may be more suitable for clinical development [143–145].

### Non-viral delivery

Recently, non-viral delivery systems have become more attractive due to their safety, low toxicity, suitability for repeat administration, and ease of scale up and manufacture. Polymer and lipid nanoparticle-based delivery systems are the two most successful platforms. Other nanoparticle systems such as magnetic nanoparticles have been used as well.

### Polymer nanoparticle platform

Polymer nanoparticles, such as Poly (lactic-co-glycolic acid) or PLGA and Polyethyleneimine (PEI), have been widely used as miRNA delivery vehicles in GBM. PLGA-nanoparticles have been used to deliver antimiR-21 and antimiR-10b into GBM cells, which led to enhanced TMZ chemosensitivity both *in vitro* and *in vivo* [146–148]. Many research reports from different groups have shown that miRNAs can be successfully delivered by PEI nanoparticles [127, 149]. By using magnetic resonance (MR)-guided focused ultrasound (FUS), miR-34a encapsulated in PEI nanoparticles was delivered across the blood brain barrier as a treatment modality for GBM [150]. By using cationic polyurethane-short branch polyethylenimine (PU-PEI), miR-145 was delivered to GBM cells in a CD133(+) immunocompromised mouse model, which cause the loss of stem cell-like properties and reduction in chemoradioresistance [125].

### Lipid nanoparticle platform

Lipid nanoparticle delivery systems have great advantages in ensuring the stability of miRNAs under physiological conditions. This property makes lipid nanoparticles a very useful carrier for miRNAs for clinical applications. Because of electrostatic interactions, positively charged lipids formulated with negatively charged miRNAs result in easy to form complexes. These complexes improve the absorption rates of miRNAs [137]. Co-delivery of pemetrexed and miR-21 antisense oligonucleotide to glioblastoma cells by cationic solid lipid nanoparticles showed high cellular uptake efficiency with low toxicity [151, 152]. By using chlorotoxin-coupled stable nucleic acid lipid particles (CTX coupled SNALPs), Costa et al. showed that systemic delivery of anti-miR-21 resulted in the reduction of proliferation, repression of tumor growth, and the enhancement of apoptosis in a GBM mouse model [92]. Further, Yaghi et al. demonstrated that lipid nanoparticles containing miR-124 prolonged survival, prevented tumor recurrence, and induced immune memory in a murine model [153]. Overall, lipid-based nanoparticle carriers have become a powerful tool for the delivery of miRNA and are likely to find broad clinical application.

### Challenges for nanoparticle delivery to glioblastoma

In order to increase drug delivery ability to GBM, most nanoparticle are designed to cross the BBB through transcytosis pathway. The nanoparticle size is one of the very important aspects that can influence their penetration into the brain. Smaller nanoparticles (<15 nm) can cross the BBB more easily through the transmembrane or the paracellular pathway [154–156]. Bigger nanoparticles (<100 nm), can cross the BBB by cell endocytosis since it is size-dependent [157–159]. Larger nanoparticles (>100 nm), if correctly designed and functionalized, are still able to cross the BBB, although to a slightly lower extent [28]. However, even if the larger size nanoparticles successfully cross the BBB, they would still have difficulty getting to tumor cells through the diffusion barrier formed by the brain extracellular space (ECS) which is a foam-like structure that connects the interstitial space between neural cells 40 nm to 700 nm in size [160–162]. Small nanoparticles are able to diffuse through the ECS providing for more efficient drug delivery. Thus, the optimal nanoparticle size is the key for drug delivery to the brain. On the other hand, nanoparticle surface can be conjugated with related ligands such as transferrin [163, 164], insulin [165, 166], glutathione [167, 168] and others to support the BBB crossing by receptor-mediated transcytosis. Furthermore, pegylation of nanoparticles extends their half-life time thus increasing their residence time in cerebral circulation and increasing their chances for delivery to the brain [169]. Recently, by combining microbubble-enhanced ultrasound technique and cationic nanoparticles, researchers temporary disrupted BBB non-invasively and effectively delivered RNA-based drugs to brain tumors [170]. Based on these examples, many promising, well-designed nanoparticle systems, capable of BBB crossing are being tested in preclinical studies. However, more studies are needed for translation of these approaches to clinical trials.

## CONCLUSIONS

Recent studies have proven the importance of miRNA and its therapeutic benefits in GBM. Because one miRNA can target multiple genes and each gene can be regulated by different miRNAs, the effects of this kind of therapy are likely to be very potent. However, thorough and careful studies need to establish the potential for off-target effects of these multipronged therapeutic agents. With time an increasing number of miRNAs and their corresponding targets are being identified and vetted for therapy. Therefore, we are beginning to see the emergence of this new class of molecular therapy as a novel and powerful treatment against multiple cancers, including GBM. Promisingly, recent reports show that miRNAs not only reactivate the immune system [171] but also overcome drug resistance [172]. In addition, miRNA-based therapies are being increasingly used in combination with conventional therapies. These advancements in our understanding of miRNA biology, together with the growth of the field of nanotechnology poised to address the critical issue of delivery, have moved us closer to the possibility for successful miRNA-based treatment for GBM.

## Abbreviations

TMZ: temozolomide
BBB: blood-brain barrier
CNS: central neuron system
ERK: extracellular signal-regulated kinase
EGFR: epidermal growth factor receptor
FAK: focal adhesion kinase
TGF-β2: transforming growth factor β2
PDGFRA: platelet-derived growth factor receptor-α
Bmi-1: B lymphoma Mo-MLV insertion region 1 homolog
RhoC: Ras homolog gene family member C
uPAR: urokinase-type plasminogen activator receptor
PUMA: p53-upregulated modulator of apoptosis
ECOP: epidermal growth factor receptor coamplified and overexpressed protein
MMP: matrix metalloproteinase
MAZ: myc-associated zinc finger protein
circRNA: circular RNAs
AAVs: adeno-associated viruses
